# Dietary supplementation of protease and organic acid in poultry by-product meal-based diet in broilers

**DOI:** 10.5713/ab.24.0136

**Published:** 2024-06-26

**Authors:** Muhammad Ahsan Yaseen, Waqar Iqbal, Shaukat Ali Bhatti, Muhammad Saif ur Rehman, Asghar Subhani, Muhammad Shoaib, Muhammad Aziz ur Rahman, Muhammad Umar Yaqoob

**Affiliations:** 1 Institute of Animal and Dairy Sciences, University of Agriculture, Faisalabad 38000, Pakistan; 2 Faculty of Veterinary Medicine, University of Teramo, Loc. Piano D’Accio, Teramo 64100, Italy; 3 Laboratory of Quality & Safety Risk Assessment for Animal Products on Feed Hazards (Beijing) of the Ministry of Agriculture & Rural Affairs, Institute of Feed Research, Chinese Academy of Agricultural Sciences, Beijing 100081, China; 4 Provincial Key Agricultural Enterprise Research Institute of King Techina, Hangzhou King Techina Feed Co., Ltd., Hangzhou 311107, China; 5 College of Animal Science, Zhejiang University, Hangzhou 310058, China

**Keywords:** Growth Performance, Gut Health, Organic Acid, Poultry By-product Meal, Protease, Serum Biochemistry

## Abstract

**Objective:**

This study investigated the impact of supplementation of protease and organic acid on growth performance and other biological parameters in broilers fed poultry by-product meal (PBM) based diet.

**Methods:**

Five hundred 1-day-old broiler chicks (Ross 308) were distributed into five treatments with 5 replicates, each pen having 20 birds, and fed each group one of five isocaloric and isonitrogenous diets in two phases: stater phase (1 to 21 days) metabolizable energy (ME) 3000 kcal/kg; crude protein (CP) 22%, and a finisher phase (22 to 35 days) ME 3,200 kcal/kg; CP 19.5%. The dietary treatments were: i) standard broiler ration (Cont); ii) The control diet with 25% of the soybean meal replaced by PBM on an equivalent protein basis (PBM); iii) PBM diet supplemented with 0.5 g/kg of protease (PBMP); iv) PBM diet supplemented with 1 g/kg organic acid (PBMO); and v) PBM diet addition with 0.5 g/kg protease and 1 g/kg organic acid (PBMPO).

**Results:**

The overall data showed that feed conversion ratio was improved (p<0.05) in the PBMP group. Apparent CP digestibility was higher (p<0.05) in both Cont and PBMP groups. Jejunal villus height increased (p<0.05) in PBMP and PBMPO groups, while only the PBMO group exhibited a higher (p<0.05) crypt depth. Lipase activity was increased (p<0.05) in the PBMP, PBMO, and PBMPO dietary treatments. However, trypsin activity showed a significant increase (p<0.05) in the PBMP and PBMO groups. Serum biochemistry increased (p<0.05) globulin and total protein levels in the PBMP group.

**Conclusion:**

PBM could partially replace the soybean meal with supplementation of either protease or organic acid in broiler diets without impairing overall growth performance. Furthermore, careful optimization must be considered when combining protease and organic acids.

## INTRODUCTION

The poultry industry has achieved tremendous advancements in broiler chicken performance and efficiency through improvements in nutrition and management. However, feed costs account for 70% to 75% of total broiler production expenses, presenting a major cost for producers [1]. Dietary protein sources are usually the most expensive feed ingredients, strongly impacting performance, feed cost, and ultimately the profitability of broiler enterprises [2]. Soybean meal (SBM) is a chief protein source due to its high crude protein (CP) (43.9% to 48.8%) level, a relatively good amino acid profile, high ileal protein digestibility coefficients (85%), and minimal nutrient composition variability to support broiler growth [3]. However, fluctuating SBM prices have necessitated evaluating economical alternative protein sources to partially replace SBM without impairing the broiler performance or profit margins [4]. Poultry by-product meal (PBM), derived from various poultry slaughter by-products like spent hens, viscera, feathers, heads, and feet, represents an abundant and cheaper protein source that can replace SBM in monogastric animal feed in many countries [5]. Despite its high CP content (58.4% to 62%), PBM has limited utilization in broiler feeds due to its variable nutrient composition, inferior protein quality, and poor digestibility (47%), attributed to differences in raw materials, processing methods, and high keratin content [6]. Thus, the determination of PBM nutrient utilization is of immense importance [7].

Previous studies found including PBM in poultry feed at 3% or 6% [8], 7.5% [9], and 10% did not adversely impact broiler performance [10]. However, levels exceeding 10% decreased growth and feed efficiency [11]. Strategies like supplementing protease enzymes and organic acids have improved PBM nutritional value by enhancing weight gain (WG), feed efficiency, protein digestibility, and livability in broilers [12]. Protease supplementation provides extracellular enzymatic activity within the intestinal lumen to hydrolyze protein fractions resistant to endogenous proteolysis and those escaping small intestine digestion, thereby increasing amino acid availability for growth and enhancing bird performance [13]. Inclusion of 3% PBM in diet along with 0.2 g/kg protease, or a protease supplemental diet (30,000 IU/kg) along with CP at 21% improved performance as well as nutrient digestibility in broilers [14]. Mono component protease at 0.2 g/kg improved the villus height (VH) and villus to crypt ratios compared to control SBM based basal diet [15]. Organic acids like lactic, fumaric, citric, and sorbic acid have antimicrobial properties, and improve gut health and digestive functions by lowering gut pH to inhibit pathogens while stimulating beneficial bacteria [16]. Along that, dietary organic acids heighten gastric proteolysis and pancreatic secretions, enhance nutrient digestibility, reduce intestinal infections, stimulate the development of intestinal villi, and improve broiler growth performance [17]. Organic acid blends have synergistic benefits over individual acids by providing multifaceted modes of action [18]. Previous studies found that supplementing broiler feeds with citric acid (0.5%) or blends of organic acids improved WG, feed efficiency, and protein digestibility [19]. As prior studies predominantly employed PBM inclusion rates, our study took a different approach by incorporating PBM as a replacement for SBM on an equivalent protein basis. This method aimed to uphold the CP level as specified by the Ross 308 guidelines. In our study, we substituted 25% of the SBM with PBM in the control diet. However, when assessed by inclusion rates, this substitution amounted to 7.73% during the starter phase (day 1 to 21) and 6.56% during the finisher phase (day 22 to 35).

However, there is limited information on combining protease and organic acid supplementation in PBM-based broiler diets. Hence, this study assessed the effects of supplementing protease and an organic acid in diet with 25% SBM replaced by PBM on broiler growth performance, digestive enzyme activities, nutrient digestibility, jejunum morphology, carcass and meat quality traits, and serum biochemistry parameters.

## MATERIALS AND METHODS

The experimental protocol for this study received ethical consent from the Institute of Animal and Dairy Sciences, University of Agriculture Faisalabad, (approval No. 20253/56, dated 2020.08.13), ensuring compliance with ethical standards for use, and welfare of animals.

### Animals and experimental design

Five hundred broiler chicks (Ross 308; day-old) were randomly distributed into five treatments with 5 replicates (20 chicks/replicate). Birds were equally placed in pens covered with 9 to 10 cm of wooden shaving floor bedding (5×5×20) in a completely randomized design. Each pen was supplied with a manual hanging feeder and drinker line for *ad libitum* approach to feed and water. The stocking density was kept at 0.65 ft2/bird throughout the 35-day trial period. Temperature for shed was set at 35°C during 1st week then steadily decreased by 5°C each week until accomplishing 21°C for the rest of study. The photoperiod was set at continuous light (24 L) and relative humidity was maintained between 60% and 70%. Biosecurity measures like thorough cleaning, disinfection and fumigation were implemented prior to housing the chicks. All birds were vaccinated against common poultry diseases according to schedule for infectious bronchitis, Newcastle disease (ND), infectious bursal disease, and ND at the age of 1, 8, 17, and 25 day, respectively.

### Experimental diets

Five isocaloric and isonitrogenous treatment diets were formulated and divided into two phases to meet or exceed Ross 308 recommendations (2019). i) Stater, day 1 to 21, metabolizable energy (ME) 3,000 kcal/kg and crude protein (CP) 22%; ii) Finisher, day 22 to 35, ME 3,200 kcal/kg and CP 19.5%. Proximate analysis was conducted on the treatment diets (Table 1). The nutrient composition of the PBM was as following: dry matter (DM) 93.0%; CP 57.0%; ether extracts 28.0%; crude fiber 8.0%; crude ash 8.0%.

The dietary treatments were: i) Corn-soy control diet (Cont); ii) The control diet with 25% of the SBM replaced by PBM on an equivalent protein basis (PBM diet); iii) PBM diet supplemented with 0.5 g/kg of protease (PBMP diet); iv) PBM diet supplemented with 1 g/kg organic acid (PBMO diet); and v) PBM diet addition with 0.5 g/kg protease and 1 g/kg organic acid (PBMPO diet). The commercial protease product CIBENZA DP100 (Novus International, Inc., St. Charles, MO, USA), containing *Bacillus licheniformis* PWD-1 fermentation solubles mixed with minced limestone and has a bottommost enzymatic activity of 600,000 U/g, and the organic acid product (EASTMAN PRO GIT SF3; Eastman Chemical Ltd., Kingsport, TN, USA), which is a mix of 16% lactic, 5% citric, 26.5% formic, and 13.1% medium chain fatty acids and 3.5% mono-, di- and triglycerides, were top-dressed during the mixing of experimental diets whose formulation was as per data of the Brazilian Tables for Poultry and Swine [6]. The feeding trial and all related analysis were performed at the Poultry Research Farm of the University of Agriculture Faisalabad, Pakistan.

### Sampling and measurements

#### Growth performance

The body weight (BW) of all chicks at day-old and weekly thereafter to check WG. The feed intake (FI) was measured weekly per pen by calculating the difference between feed fed offered and refusal. The feed conversion ratio (FCR) was determined for each period and overall using FI and WG data.

#### Digestibility trial

For digestibility assay, the acid insoluble ash (AIA), in the form of Celite, as an indigestible marker at a rate of 1%, was mixed to all the experimental diets on d 32 and fed to broilers until day 35 for the adaptation period. Excreta samples were collected twice on d 35, within polythene zip bags from each pen, pooled, and kept at −20°C until further analysis. This resulted in 5 replicate samples per treatment group. Feed samples from each treatment diet were also collected. For laboratory analysis, fecal samples dried out at 57°C for 72 hours, ground, and passed through a one-mm screen [20]. Both feed and excreta samples were analyzed for DM and CP contents using the AOAC method [21], while AIA was determined in the ashed samples of diet and digesta [22].

#### Digestive enzyme activity

On the 35th day, two birds were randomly picked from each pen and euthanized by severed neck cut to cut the jugular vein and carotid artery to collect jejunum. Amylase, lipase and trypsin activities in jejunum were determine using commercial kits (Nanjing Jiancheng Bioengineering Institute, Nanjing, China) according to the the instructions of manufacturer.

#### Intestinal morphology

On the 35th day, two birds for each pen (10 birds/treatment group) were randomly chosen and euthanized. The jejunum was located, and samples were collected from the midpoint region. Approximately 2 cm segments were gently flushed with saline to remove any digesta particles. These samples were immediately fixed in a 10% neutral buffered formalin solution and processed according to the method described by Xu et al [23]. Using a Nikon phase-contrast microscope connected to a MicroComp integrated digital image analysis system (Nikon Eclipse 80i, Nikon, Tokyo, Japan), VH, crypt depth (CD), and the VH to CD ratio were calculated. Only intact villi, extended from tip to base, were selected for measurement. VH was calculated from the tip to the junction of villus crypt, while CD was specified as the depth of the invagination between adjacent villi. At least 10 well-oriented, intact villi and associated crypts were measured per sample. The VH and CD values were then averaged to obtain a single representative value per bird. The VH:CD ratio was calculated based on the average VH and CD measures. Since the protease used (Cibenza DP100; Novus International, Inc., USA) is an alkaline protein, it is most active in the lower small intestine; the jejunum and ileum, and least active in the duodenum, therefore the jejunum was selected as a point to measure VH and CD where the effect of enzyme could be quantified [24].

#### Carcass characteristics and meat quality analysis

On the 35th day, two birds for each pen (10 birds/treatment group) were picked, then carefully weighed and humanely slaughtered by making a precise cut to sever the neck at the jugular vein and carotid artery to assess carcass and meat quality traits. After complete bleeding out, the birds were defeathered and eviscerated. The neck, head, legs, and viscera were eliminated to obtain hot eviscerated carcass weight. The carcass, breast and thigh meat, heart, liver, spleen, gizzard, and abdominal fat pad were weighed separately. The weights were stated as percentages of the eviscerated carcass weight to determine dressing percentage and relative organ weights.

Breast meat (pectoralis major) and thigh meat samples were collected to assess meat quality traits. The pH was measured by homogenizing (VELP, Scientifica, Usmate Velate, Italy) about 2 g meat of each sample in distilled water (10 mL) followed by direct pH measurement using digital pH meter (HI 99163) from Hanna Instruments Inc., Woonsocket, RI, USA. Water holding capacity (WHC) was evaluated for the breast and thigh meat samples utilizing the compression technique. Approximately 15 g of meat was weighed, placed in a tube with filter paper and centrifuged for 15 minutes at 5,000 rpm (Eppendorf Centrifuge5804R; Eppendorf, Hamburg, Germany) and 4°C. The WHC was computed from the difference between initial sample weight and centrifugal moisture loss [24].

#### Serum biochemistry analysis

On the 35th day, two birds for each pen (10 birds/treatment group) were chosen at random, slaughtered, and blood samples were promptly collected from the jugular vein into tubes without anticoagulant. The samples were stayed at room temperature for 120 minutes to let the clotting, then centrifuged was done to obtain supernatant serum from these samples (Beckman Coulter Allegra 6R Refrigerated Centrifuge; Beckman Coulter, Inc., Brea, CA, USA) at 6,000 rpm for 10 minutes. The clear non-hemolyzed sera were aspirated and put in storage at −20°C till analysis. Serum samples were analyzed for the following biochemical parameters using commercial assay kits (Spinreact, Girona, Spain): albumin (ALB), globulin (GLB), total protein (TP), albumin to globulin ratio (ALB:GLB), aspartate aminotransferase (AST), alanine aminotransferase (ALT), and uric acid (UA) by automated chemistry analyzer (Mindray BS-240; Mindray Medical International Ltd. Shenzhen, China).

### Statistical analysis

Research data was analyzed statistically with analysis of variance in SPSS 22.0. The experimental design was completely randomized. The main effects of the dietary treatments on each parameter were analyzed. Mean values between treatments were compared using Tukey’s post hoc multiple comparisons test to find significant differences at p<0.05 level.

## RESULTS

### Growth performance

Throughout the starter phase (day 1 to 21), broilers that were fed the Cont, PBM, and PBMP diets explained significantly higher WG (p<0.05) contracted to the other treatment groups. Conversely, WG was lowest (p<0.05) in PBMO diet group. A similar trend was observed in FI, with the Cont and PBM diets resulting in significantly higher FI (p<0.05) compared to the other diets. In the finisher phase (day 22 to 35). No significant results were observed (p>0.05) in WG, FI, and FCR on all dietary treatments. Throughout the overall experimental period (day 1 to 35), WG and FI were similar (p<0.05) across all treatment groups. However, the FCR was notably improved (p<0.05) with the PBMP diet compared to control diet in broilers. In summary, replacing 25% of SBM with PBM did not adversely affect overall growth performance. Furthermore, supplementation with protease, but not organic acid, significantly enhanced feed efficiency in PBM-based diets (Table 2).

### Nutrient digestibility

The apparent digestibility of DM was statistically similar across all dietary treatments (p>0.05), ranging from 91.20% in the organic acid supplemented PBMO diet to 93.14% in the control group. However, the CP digestibility was highest (p<0.05) in the protease supplemented PBMP diet (76.34%) and control diet (73.41%). On the other hand, CP digestibility was lowest (p<0.05) in the combined protease and organic acid supplemented PBMPO diet (65.64%). In conclusion, supplementing protease in PBM-based broiler diets enhanced the utilization of dietary protein, as evidenced by the significantly higher CP digestibility compared to the PBM diet alone. Organic acids did not improve protein digestibility and lowered CP digestibility when combined with protease (Table 3).

### Digestive enzyme activity

Non-significant results (p>0.05) were observed in amylase activity on all dietary treatments from 2.00 to 2.11 U/mg protein. However, lipase activity improved (p<0.05) with the supplementation of protease (PBMP), organic acids (PBMO), or both (PBMPO) contrasted to the Cont. The highest lipase of 31.36 U/mg occurred with organic acid addition (PBMO). Furthermore, Trypsin activity significantly increased (p<0.05) with protease (230.00 U/mg protein) or organic acid (227.00 U/mg protein) supplementation compared to control (212.60 U/mg protein) and PBM alone (199.80 U/mg protein). In conclusion, the supplementation of protease or organic acids to PBM-based diets enhanced the digestive capacity of broiler intestines by increasing lipase and trypsin activities (Table 4).

### Jejunal morphology

The Cont, PBMP, and PBMO diets resulted in significantly decreased VH (p>0.05) compared to others. The PBM diet significantly reduced (p<0.05) the jejunum VH from 1,183.80 μm in control to 1,025.80 μm. Supplementing protease (PBMP) or organic acids (PBMO) to the PBM diet restored the VH to levels like the control, at 1,189.80 and 1,193.00 μm respectively. Supplementation of the PBM-based diet with organic acid (PBMO) elicited a significant increase (p<0.05) in jejunal CD, with a mean value of 114.00 μm compared to 88.60 μm in birds fed only PBM diet. Dietary addition of protease (PBMP, 101.60 μm) or the combination treatment (PBMPO, 96.00 μm) did not impact CD relative to control (103.80 μm). Non-significant results were observed (p>0.05) in VH:CD ration on all dietary treatments. In conclusion, the reduced jejunal VH indicates the negative effects of dietary PBM on gut morphology in broilers. However, supplementing protease or organic acids appears to counteract these adverse effects by improving villus development (Table 5).

### Carcass characteristics and meat quality

The carcass characteristics together with dressing percentage, relative breast and thigh meat weight, and relative weight of gizzard, heart, liver, spleen, and abdominal fat were statistically similar (p>0.05) across all dietary treatments. No significant differences (p>0.05) were observed in WHC and pH for breast meat quality in all dietary treatments. The pH values ranged from 5.92 in the protease supplemented PBM diet (PBMP) to 6.08 in the combined protease and organic acid supplemented PBM diet (PBMPO). The breast meat WHC was numerically higher in PBMP (57.82%) compared to the other treatments. Similarly, the thigh meat pH and WHC were statistically similar (p>0.05) between dietary treatments. The pH ranged from 6.18 to 6.37, with PBM having the highest thigh meat pH numerically. The thigh meat WHC was numerically highest in PBMPO (51.71%), compared to 42.40% to 49.42% in the other treatments. In summary, the replacement of SBM with PBM, with or without protease and organic acid supplementation, did not significantly influence the carcass characteristics and meat quality parameters measured in this study (Table 6).

### Serum biochemistry

Of the serum biochemical parameters measured, only TP and GLB levels significantly affected (p<0.05) across all dietary groups. PBMP diet showed a significant improvement (p<0.05) in circulating GLB and TB levels relative to control and PBM groups, with mean values of 7.60 and 6.83 g/dL respectively. The results of other serum biomarkers including liver enzymes ALT, AST, ALB, ALB:GLB ratio, and UA were non-significant (p>0.05) across dietary treatments. In summary, supplementing protease in PBM-based broiler diets significantly increased TP and GLB levels, indicating an improvement in protein status (Table 7).

## DISCUSSION

This study demonstrated that replacing SBM with PBM up to 25%, does not negatively impact overall growth performance broiler. Broilers fed with a diet substituting 25% of SBM with PBM exhibited similar (p>0.05) WG and FI compared to those on a basal (corn-soy) diet over the 35-day trial period. This finding is consistent with prior research that found no adverse effects on broiler performance when incorporating PBM at various levels (3%, 6%, 9%, or 10%) in the diets [25]. During starter phase lower FI and WG was observed in the organic acid diet (PBMO) group could be attributed to several factors. Organic acids are known to lower feed palatability due to their sour taste, which can reduce voluntary FI, especially in young birds that are more sensitive to changes in feed flavor. Additionally, the inclusion level of organic acids might have caused an initial adaptation period where the birds' gastrointestinal tract adjusted to the new diet, temporarily affecting FI and growth performance. However, this study revealed a better FCR (p<0.05) in broiler group fed PBMP or protease-supplemented diet compared to the control group (1.63 vs 1.67) during overall growth period (day 1 to 35). These results are in line with Mahmood et al [26] who observed better (p<0.05) FCR and non-significant results (p>0.05) on FI and body WG in broilers with 0.2 g/kg protease supplementation with various levels of PBM-based diets.

Similarly, Gwak et al [24] reported that broiler groups fed with enzyme (alkaline protease) hydrolyzed PBM, with an activity of 200,000 U/g, displayed significantly higher FCR compared to the control SBM and PBM groups. However, this study indicated poor FCR (p<0.05) in broiler groups fed PBM and PBMPO diets, possibly due to the highly variable nutritional composition and suboptimal protein quality of PBM, attributed to its high keratin content which limits PBM digestibility and amino acid availability [27]. The minimal (p<0.05) CP digestibility in broiler groups fed the PBMPO and PBM diets support this concern. Nonetheless, supplementing PBM diet at 0.5 g/kg of protease (Cibenza DP100; Novus International, Inc., USA) significantly improved CP digestibility, matching that of the control diet (76.34 vs 73.41). Proteases likely enhanced protein utilization by hydrolyzing keratin and other antinutritional factors resistant to endogenous enzymatic breakdown [13]. This study supported by Xu et al [28] who found that adding keratinase at 200,000 U/kg improved nutrient utilization and performance in broilers fed feather meal-based diets, while Freitas et al [29] confirmed that multienzyme complexes enhance protein digestibility, corroborating our findings.

However, in this study, supplementation of PBM diet with organic acid at a rate of 1 g/kg of feed did not influence CP digestibility. Conversely, supplementing the PBM diet with both organic acid and protease significantly lowered (p<0.05) the CP digestibility compared to PBM diets supplemented with or without protease alone and the control diet. Organic acids may have interacted with the alkaline protease and partially inhibited its activity and effectiveness by Adil et al [17].

Moreover, this study indicated that supplementation of protease or organic acid in PBM based diets did not significantly influence amylase activities (p>0.05) but improved (p<0.05) lipase and trypsin activities in broiler groups fed PBMP, PBMO, PBMPO, and PBMP, PBMO diets respectively, providing a mechanistic explanation for improved protein digestion compared with control group. This demonstrates the value of exogenous protease and organic acid for unlocking the nutritional potential of lower quality protein sources like PBM in broiler diets. Nonetheless, organic acid improved lipase and trypsin levels, which likely contributed to the numerically improved FCR (1.66 vs 1.70) and CP digestibility (70.25 vs 67.52) in PBMO group when compared with alone PBM group. Organic acids optimize conditions for endogenous enzymes and suppress pathogens by lowering gut pH that compete with the host for nutrients [17]. Present results are reliable with some reports that using organic acids in broiler improves digestive enzyme activity [30]. The lower trypsin activity in the PBMPO group compared to the either PBMP or PBMO group could be attributed to the potential antagonistic interaction between protease and organic acid. Organic acids can lower the pH in the gastrointestinal tract, which might not be the optimal condition for exogenous protease activity. This sub-optimal condition can inhibit the activation and functioning of both endogenous and exogenous enzymes, including trypsin. Furthermore, combining both additives might have led to competitive inhibition, reducing overall enzyme activity.

Digestion and absorption of nutrients takes place chiefly in the small intestine. Improved feed efficiency in poultry can be partially justified by improved gut morphology to enhance its ability to absorb nutrients. But the PBM diet had detrimental impacts on gut morphology as compared to control, indicated by decreased VH (1,025.80 vs 1,183.80 μm) and CD (88.60 vs 103.80 μm) in the jejunum. This likely impaired nutrient absorption. However, supplementing PBM with protease or organic acids counteracted these negative effects and improved VH to match the control diet. The structural enhancements suggest increased absorptive capacity, which improves growth efficiency. Organic acids may stimulate villus development by reducing inflammation and cellular damage associated with high pathogen loads [19]. The deeper crypts may be explained by diet-induced changes in microbial populations. However, VH:CD ratio remained (p>0.05) unaffected by all treatments.

Carcass traits such as dressing percentage and carcass yield, muscle pH, and WHC of thigh and breast meat were not influenced (p>0.05) by various dietary treatments in this study, consistent with past findings that organic acids or protease supplementation in PBM based diets do not alter these parameters [31]. Similarly, Çenesiz et al [32] claimed non-significant (p>0.05) effects of organic acid addition in PBM diet on pH of breast meat and WHC and in broilers. From blood serum biochemical analysis, only the levels of TP and GLB were better (p<0.05) with PBMP diet compared to control and PBM diet groups, implying benefits to protein nutritional status from enhanced digestion and amino acid absorption. This finding aligns with studies reporting increased serum TP and GLB levels with supplementation of protease broilers diet [33]. Other serum biomarkers like ALT, AST, ALB, ALB:GLB, and UA did not influence (p> 0.05) by various treatments. Elevated blood GLB may indicate stimulated antibody production to counteract immune challenges from antigenic compounds in PBM. Overall, protease addition enhanced the protein nutritional status of broilers fed PBM-based diets, while performance parameters remained unaffected. In contrast, Hafeez et al [34] claimed non-significant effect of protease addition and phytogenetics on blood TP levels in broilers, which might be due to different sources of protease used in these experiments or different experimental designs.

## CONCLUSION

The present study demonstrated that SBM could be partially replaced by PBM with supplementation of either protease or organic acid in broiler diets without compromising overall growth performance. However, careful optimization must be considered when combining protease and organic acids. Their complex interactions may significantly impact overall performance, underscoring the need for precision in our work. Future research should focus on fine-tuning these combinations to maximize their synergistic benefits.

## Figures and Tables

**Table 1:** Ingredients and nutrients composition of experimental diets

Items	Starter phase (d 1 to 2^1)^		Finisher phase (d 22 to 35)	
	
	Cont^1)^	PBM^1)^	Cont^1)^	PBM^1)^
Ingredients				
Corn	52.73	59.32	57.68	63.28
Soybean meal 45%	38.89	27.82	33.04	23.65
Poultry byproduct meal	0.00	7.73	0.00	6.56
Soybean oil	3.58	1.28	5.64	3.68
Calcium carbonate	0.90	0.68	0.73	0.54
Dicalcium phosphate	2.17	1.18	1.78	0.94
Sodium chloride	0.39	0.24	0.39	0.26
Sodium bicarbonate	0.30	0.60	0.12	0.38
L-Lysine sulphate	0.36	0.47	0.15	0.25
DL-Methionine	0.37	0.36	0.27	0.26
L-Threonine	0.11	0.12	0.00	0.00
Choline chloride	0.10	0.10	0.10	0.10
Vit. Min. premix^2)^ (g/kg)	0.10	0.10	0.10	0.10
Total	100.00	100.00	100.00	100.00
Nutrients composition, % (Calculated)				
Metabolizable energy (Kcal/kg)	3,000.00	3,000.00	3,200.00	3,200.00
Crude protein	22.00	22.00	19.50	19.50
Ether extract	5.8	5.58	7.97	7.78
Crude fiber	2.94	2.54	2.76	2.42
Ash	4.92	4.62	4.34	4.08
Dig. Lysine	1.28	1.28	1.03	1.03
Dig. Methionine	0.67	0.67	0.54	0.55
Dig. Threonine	0.86	0.86	0.67	0.67
Nutrients composition, %^3)^ (Analyzed)				
Dry matter	90.23	90.43	90.54	90.34
Crude protein	21.98	22.01	19.67	19.5
Ether extract	5.50	5.65	7.98	8.04
Acid insoluble ash^4)^	1.23	1.43	1.10	1.15

1) Cont, standard broiler ration; PBM, control diet with 25% of the soybean meal replaced by poultry by-product meal (PBM) on an equivalent protein basis; PBMP, PBM diet supplemented with 0.5 g/kg of protease; PBMO, PBM diet supplemented with 1 g/kg organic acid; PBMPO, PBM diet supplemented with 0.5 g/kg protease and 1 g/kg organic acid.

2) Vitalink® is a vitamins premix; each kg of it supplied the following: vitamin A 20,000 KIU; vitamin D_3_ 5,400 KIU; vitamin E 48,000 mg; vitamin K_3_ 4,000 mg; vitamin B_1_ 4,000 mg; vitamin B_2_ 9,000 mg; vitamin B_6_ 7,600 mg; vitamin B_12_ 20 mg; Niacin 60,000 mg; Pantothenic acid 20,000 mg; Folic acid 1,600 mg; Biotin 200 mg.

3) Nutrimin is a minerals premix; each Kg of it supplied the following: Iron 10,000 mg; Zinc 120,000 mg; Manganese 140,000 mg; Copper 12,000 mg; Iodine 1,800 mg; Cobalt 400 mg; and Selenium 360 mg.

4) Analysis of acid insoluble ash was conducted after supplementation with Celite.

**Table 2:** Effect of protease and organic acids supplementation on growth performance in broilers fed poultry by-product meal based diet

Parameters	Treatments^1)^					SEM	p-value

	Cont	PBM	PBMP	PBMO	PBMPO		
Starter phase (d 1 to 2^1)^							
Weight gain (g)	942.7^a^	938.2^a^	930.0^a^	870.1^b^	891.8^ab^	14.29	0.031
Feed intake (g)	1,271.0^a^	1,294.1^a^	1,238.3^ab^	1,174.5^b^	1,238.4^ab^	20.16	0.013
FCR (g/g)	1.35	1.38	1.33	1.35	1.39	0.01	0.462
Finisher phase (d 22 to 35)							
Weight gain (g)	1,056.4	1,009.1	998.6	1,028	950.8	17.47	0.531
Feed intake (g)	1,897.3	2,008.2	1,923.3	1,970	1,885.5	22.99	0.563
FCR (g/g)	1.81	2.01	1.93	1.92	1.98	0.03	0.226
Overall period (d 1 to 35)							
Weight gain (g)	1,979.1	1,947.3	1,928.6	1,898.1	1,842.6	23.22	0.249
Feed intake (g)	3,298.3	3,302.3	3,141.6	3,144.5	3,123.9	40.24	0.412
FCR (g/g)	1.67^ab^	1.70^a^	1.63^b^	1.66^ab^	1.70^a^	0.01	0.043

SEM, standard error of the mean; FCR, feed conversion ratio.

1) Cont, standard broiler ration; PBM, control diet with 25% of the soybean meal replaced by poultry by-product meal (PBM) on an equivalent protein basis; PBMP, PBM diet supplemented with 0.5 g/kg of protease; PBMO, PBM diet supplemented with 1 g/kg organic acid; PBMPO, PBM diet supplemented with 0.5 g/kg protease and 1 g/kg organic acid.

a,b Values within the same row with different superscripts, differ significantly (p<0.05).

**Table 3:** Effect of protease and organic acids supplementation on nutrient digestibility in broilers fed poultry by-product meal based diet

Parameters (%)	Treatments^1)^					SEM	p-value

	Cont	PBM	PBMP	PBMO	PBMPO		
Dry matter	93.14	92.89	91.72	91.20	91.50	0.39	0.768
Crude protein	73.41^a^	67.52^b^	76.34^a^	70.25^ab^	65.64^b^	1.94	0.032

SEM, standard error of the mean.

1) Cont, standard broiler ration; PBM, control diet with 25% of the soybean meal replaced by poultry by-product meal (PBM) on an equivalent protein basis; PBMP, PBM diet supplemented with 0.5 g/kg of protease; PBMO, PBM diet supplemented with 1 g/kg organic acid; PBMPO, PBM diet supplemented with 0.5 g/kg protease and 1 g/kg organic acid.

a,b Values within the same row with different superscripts differ significantly (p<0.05).

**Table 4:** Effect of protease and organic acids supplementation on digestive enzyme activity in jejunum of broilers fed poultry by-product meal based diet

Parameters (U/mg protein)	Treatment^1)^					SEM	p-value

	Cont	PBM	PBMP	PBMO	PBMPO		
Amylase	2.05	2.02	2.10	2.0	2.09	0.02	0.771
Lipase (U/g protein)	29.61^b^	29.07^ab^	30.55^a^	31.36^a^	30.38^a^	0.40	0.002
Trypsin	212.60^b^	199.80^b^	230.00^a^	227.00^a^	207.40^b^	5.76	0.001

SEM, standard error of the mean.

1) Cont, standard broiler ration; PBM, control diet with 25% of the soybean meal replaced by poultry by-product meal (PBM) on an equivalent protein basis; PBMP, PBM diet supplemented with 0.5 g/kg of protease; PBMO, PBM diet supplemented with 1 g/kg organic acid; PBMPO, PBM diet supplemented with 0.5 g/kg protease and 1 g/kg organic acid.

a,b Values within the same row with different superscripts differ significantly (p<0.05).

**Table 5:** Effect of protease and organic acids supplementation on jejunal morphology in broilers fed poultry by-product meal based diet

Parameters (μm)	Treatment^1)^					SEM	p-value

	Cont	PBM	PBMP	PBMO	PBMPO		
VH	1,183.80^a^	1,025.80^b^	1,189.80^a^	1,193.00^a^	1,129.20^ab^	31.84	0.010
CD	103.80^ab^	88.60^b^	101.60^ab^	114.00^a^	96.00^ab^	4.22	0.023
VH:CD	11.41	11.62	11.88	10.51	11.91	0.26	0.345

SEM, standard error of the mean; VH, villus height; CD, crypt depth.

1) Cont, standard broiler ration; PBM, control diet with 25% of the soybean meal replaced by poultry by-product meal (PBM) on an equivalent protein basis; PBMP, PBM diet supplemented with 0.5 g/kg of protease; PBMO, PBM diet supplemented with 1 g/kg organic acid; PBMPO, PBM diet supplemented with 0.5 g/kg protease and 1 g/kg organic acid.

a,b Values within the same row with different superscripts differ significantly (p<0.05).

**Table 6:** Effect of protease and organic acids supplementation on carcass characteristics and meat quality in broilers fed poultry by-product meal based diet

Parameters (%)	Treatments^1)^					SEM	p-value

	Cont	PBM	PBMP	PBMO	PBMPO		
Carcass characteristics							
Dressing %	62.90	62.30	61.50	61.31	59.80	0.52	0.123
Breast meat	62.03	60.51	61.11	60.90	60.21	0.31	0.514
Thigh meat	38.01	39.50	38.90	39.11	39.80	0.31	0.512
Gizzard	2.10	2.20	2.10	2.40	2.30	0.06	0.717
Heart	0.72	0.70	0.71	0.77	0.71	0.01	0.845
Liver	3.03	3.27	2.94	3.24	3.71	0.13	0.083
Spleen	0.17	0.17	0.15	0.16	0.18	0.01	0.105
Abdominal fat	2.31	3.27	2.99	3.64	3.42	0.23	0.068
Meat quality							
Breast meat							
pH	5.99	5.97	5.92	6.05	6.08	0.03	0.121
WHC (%)	45.46	49.44	57.82	49.66	49.79	2.02	0.071
Thigh meat							
pH	6.19	6.37	6.20	6.18	6.20	0.04	0.432
WHC (%)	42.77	42.4	42.53	49.42	51.71	1.99	0.164

SEM, standard error of the mean; WHC, water holding capacity.

1) Cont, standard broiler ration; PBM, control diet with 25% of the soybean meal replaced by poultry by-product meal (PBM) on an equivalent protein basis; PBMP, PBM diet supplemented with 0.5 g/kg of protease; PBMO, PBM diet supplemented with 1 g/kg organic acid; PBMPO, PBM diet supplemented with 0.5 g/kg protease and 1 g/kg organic acid.

**Table 7:** Effect of protease and organic acids supplementation on serum biochemistry in broilers fed poultry by-product meal based diet

Parameters	Treatments^1)^					SEM	p-value

	Cont	PBM	PBMP	PBMO	PBMPO		
ALT (U/L)	42.50	72.50	42.00	24.30	16.50	9.65	0.562
AST (U/L)	379.00	301.50	259.00	249.70	256.00	24.28	0.214
TP (g/dL)	3.80^b^	5.10^ab^	7.60^a^	5.23^ab^	4.45^b^	0.64	0.038
ALB (g/dL)	1.00	0.81	0.77	0.80	0.84	0.04	0.803
GLB (g/dL)	2.80^b^	4.29^ab^	6.83^a^	4.43^ab^	3.61^ab^	0.67	0.042
ALB:GLB	0.38	0.20	0.11	0.19	0.24	0.04	0.257
UA (mg/dL)	6.66	7.25	9.40	6.61	7.68	0.51	0.919

ALT, alanine aminotransferase; AST, aspartate aminotransferase; TP, total protein; ALB, albumin; GLB, globulin; ALB:GLB, albumin:globulin; UA, uric acid.

1) Cont, standard broiler ration; PBM, control diet with 25% of the soybean meal replaced by poultry by-product meal (PBM) on an equivalent protein basis; PBMP, PBM diet supplemented with 0.5 g/kg of protease; PBMO, PBM diet supplemented with 1 g/kg organic acid; PBMPO, PBM diet supplemented with 0.5 g/kg protease and 1 g/kg organic acid.

a,b Values within the same row with different superscripts differ significantly (p<0.05).
